# Debakey Forceps Crushing Technique for Hepatic Parenchymal Transection in Liver Surgery: A Review of 100 Cases and Ergonomic Advantages

**DOI:** 10.1155/2014/861829

**Published:** 2014-06-09

**Authors:** Sundeep Jain, Bharat Sharma, Mitesh Kaushik, Lokendra Jain

**Affiliations:** ^1^Department of Gastrointestinal Hepatopancreatobiliary Minimal Access & Bariatric Surgery, Fortis Escorts Hospital, Jawahar Lal Nehru Marg, Malviya Nagar, Jaipur, Rajasthan 302017, India; ^2^Department of General & Minimal Access Surgery, Soni Manipal Hospital, Sikar Road, Vidhyadhar Nagar, Jaipur, Rajasthan 302013, India

## Abstract

*Introduction and Objective*. Bleeding is an important complication in liver transections. To determine the safety and efficacy of Debakey forceps for liver parenchymal transection and its ergonomic advantages over clamp crushing method we analysed our data. *Methods*. We used Debakey crushing technique in 100 liver resections and analysed data for transection time, transfusion rate, morbidity, mortality, hospital stay, influence of different types of liver conditions, and ergonomi features of Debakey forceps. *Results*. Mean age, transection time and hospital stay of 100 patients were 52.38 ± 17.44 years, 63.36 ± 33.4 minutes, and 10.27 ± 5.7 days. Transection time, and hospital stay in patients with cirrhotic liver (130.4 ± 44.4 mins, 14.6 ± 5.5 days) and cholestatic liver (75.8 ± 19.7 mins, 16.5 ± 5.1 days) were significantly greater than in patients with normal liver (48.1 ± 20.1 mins, 6.7 ± 1.8 days) (*P* < 0.01). Transection time improved significantly with experience (first fifty versus second fifty cases—70.2 ± 31.1 mins versus 56.5 ± 34.5 mins, *P* < 0.04). Qualitative evaluation revealed that Debakey forceps had ergonomic advantages over Kelly clamp. *Conclusions*. Debakey forceps crushing technique is safe and effective for liver parenchymal transection in all kinds of liver. Transection time improves with surgeon's experience. It has ergonomic advantages over Kelly clamp and is a better choice for liver transection.

## 1. Introduction

Prevention of blood loss is a major concern during liver resections as it is the major determinant of operative outcome. Bleeding along with bile leak and hepatic failure is one of the major postoperative complications following liver resection [1–3]. Most blood loss occurs during the parenchymal transection of liver. Many methods have been introduced to achieve safe parenchymal transection. In 1958 Lin et al. introduced the finger fracture technique which involves crushing of liver parenchyma by surgeon's finger under inflow occlusion so as to isolate vessels and bile ducts for ligation [4]. This technique was subsequently improved through the use of small Kelly clamp for blunt dissection which gives better control, namely, clamp crushing or Kellyclasia [5–7]. People have also used finer versions of clamps similar to Kelly like Pean, Halstead, Heiss, or Bengolea clamps [6, 8].

Recently, many devices have been introduced for parenchymal transection. These include ultrasonic dissector, harmonic scalpel, LigaSure, dissecting sealer using radiofrequency, and staplers [9–11]. However, the clamp crushing technique is the most widely used method [3, 9, 12–14] and has multiple advantages over other more advanced methods including safety, speed, and cost-effectiveness [9].

Thumb forceps including Debakey forceps has significant advantages in terms of its design and ergonomics over Kelly clamp which were reported previously when compared for their usage for diathermy [15]. But so far its use in liver parenchymal transection has not been tried or reported in literature.

We have been using Debakey forceps for liver transections in all our liver resections for the past 8 years. The purpose of this study is to present our experience of 100 consecutive elective liver resections with Debakey forceps crushing technique. We compared the outcome after liver transection on different types of liver parenchyma—normal livers, cirrhotic livers, postchemotherapy livers, and cholestatic livers. We also highlight its ergonomic advantages over clamp crushing method. To the best of our knowledge this is the first such study reporting the usage of Debakey forceps for hepatic parenchymal transection.

## 2. Methods

This is a retrospective study of prospectively collected data of consecutive liver resections. During the period of January 2006 to October 2013 we performed a total of 146 liver resections in three hospitals under supervision of the main author (Sundeep Jain). Of these 46 were performed in emergency setting (trauma—*n* = 44; liver necrosis—*n* = 2) and excluded from the present study. Data of remaining 100 patients are presented (Table 1). Types of liver resection, according to Brisbane terminology [16], in these 100 patients are presented in Table 2.

These patients were classified in 4 groups according to the type of underlying liver parenchyma into group A—normal liver; group B—cirrhotic liver; group C—postchemotherapy liver; and group D—cholestatic liver. These four groups were compared in terms of age, gender, comorbid conditions, transection time, total operative time, postoperative length of hospital stay, blood transfusion rates, morbidity, and mortality to evaluate the effect of type of liver parenchyma with use of Debakey forceps crushing technique for liver parenchyma transection.

The first 50 (group 1) and the last 50 (group 2) were compared to evaluate the duration of transection time, total operative time, and postoperative length of hospital stay.

### 2.1. Qualitative Ergonomic Evaluation

The design along with mechanism of functioning of Debakey forceps and Kelly clamps was studied and compared using photographs taken during operation. This was to ascertain the advantages of one over the other in terms of ease of usage and the versatility of the instruments. Also the ergonomic differences in the wrist joint were studied, with the help of photographs while using Debakey forceps and Kelly clamps for liver parenchymal transection.

### 2.2. Anaesthesia Details

All the patients were induced with fentanyl 2 *μ*gm/kg and propofol 2-3 mg/kg of body weight and intubated with atracurium 0.5 mg/kg of body weight. Maintenance of anaesthesia was achieved using sevoflurane in an air-oxygen mixture with supplemental fentanyl. After induction, central venous catheterization was done uniformly in right internal jugular vein for central venous pressure (CVP) monitoring with the aim of keeping CVP less than 5 mmHg and as close to 0 mmHg as possible, during parenchymal transection. This was achieved by fluid restriction and diuretics (frusemide) in 0.5–1 mg/kg IV dose. In 8/100 patients we had to use nitroglycerine to reduce CVP to the desired levels. During this phase urine output and mean arterial pressures were maintained at more than 0.5 mL/kg/hr and more than 70 mmHg, respectively. This was done by 100–200 mL bolus fluid challenge and norepinephrine infusion at 0.05–0.1 *μ*gm/kg/min. During the low CVP stage patients were kept in head-low position to prevent the risk of air embolism. Euvolemia was finally achieved after transection and hemostasis were completed.

After the surgery all patients are reversed with neostigmine 40–80 *μ*gm/kg along with glycopyrrolate 10 *μ*gm/kg.

### 2.3. Surgical Details

All the patients with malignant conditions initially had staging laparoscopy. The abdomen was explored by either bilateral subcostal or triradiate incisions depending on the site and size of the lesion. The falciform ligament was then divided and the lobe to be resected was mobilized from surrounding attachments and structures like diaphragm and vena cava. Only in two patients (both with hepatocellular carcinoma) undergoing right hemihepatectomy, Pringle's manoeuvre was used to facilitate removal of associated portal vein tumour thrombus in one and due to excessive bleeding in another. In all patients during parenchymal transection low central venous pressure (0–5 mmHg) with head-low position was maintained.

The liver parenchymal transection was started with the marking of the line of resection using monopolar electrocautery followed by cutting the parenchyma for 2–4 mm deep. Then the parenchyma was crushed using fine tip (1 mm), 8 cm long straight or 9 cm long angled Debakey forceps depending on the depth of transection followed by coagulation of the small vessels of <2 mm size using monopolar electrocautery and ligation of the biliary and larger vascular pedicles using 2–0/3–0 silk sutures. Lastly, the biliary duct was isolated and divided in appropriate cases. Once the specimen was out the haemostasis was achieved using spray cautery and fine (3–0/4–0) prolene sutures. The bile leaks were looked for and suture ligated. The Roux-en-y bilioenteric anastomosis was done with the bile ducts of the remaining lobe wherever indicated. Prophylactic drains were placed in all the patients.

### 2.4. Statistical Analyses

Descriptive statistics are presented. All the data were computerised and analysed using STATA 11 statistical software. Intergroup comparisons were performed using Group A as control. Numerical variables have been compared using *t*-test and categorical variables using Chi-square test. *P* value <0.05 is considered statistically significant.

## 3. Results

During the study period of January 2006 to October 2013 a total of 100 elective liver resections were performed for various indications using Debakey thumb forceps for the liver parenchymal transection. There were 39 females and 61 males with a mean age of 52.4 ± 17 years. Indications and the type of liver resections performed are mentioned in Tables 1 and 2. Various comorbidities included hypertension (*n* = 11), diabetes (*n* = 8), and chronic obstructive pulmonary disease (COPD) (*n* = 5), while none had coronary artery disease. Group B had 8 patients, group C had 14 patients, and group D had 25 patients while normal liver parenchyma was in 53 patients.

Majority of patients underwent liver resection for malignant diseases (*n* = 73). Of these 14 (19%) had undergone preoperative chemotherapy (hepatoblastoma 3, gastrointestinal stromal tumor 4, and colorectal cancers 7). Obstructive jaundice was in 25/100 patients (12 gallbladder cancer, 9 hilar cholangiocarcinoma, and 1 each of hydatid disease, recurrent pyogenic cholangitis and strictured hepaticojejunostomy with right lobe atrophy and recurrent cholangitis). Seven of these 25 (gallbladder cancer 4, hilar cholangiocarcinoma 2, and hydatid cyst 1) had plastic stent placed in the common bile duct. Roux-en-y bilioenteric anastomosis was done in 22/25 patients. All the eight patients of hepatocellular carcinoma (HCC) had cirrhosis of liver due to alcohol in two, hepatitis B virus in four, and hepatitis C virus in two patients. All of these were in Child's A status without any history of decompensation in the past. None of them were under consideration for transplant. Pringle's manoeuvre was used in two patients, both with HCC (alcoholic & hepatitis C related cirrhosis).

The mean age, transection time, total operative time, and postoperative length of hospital stay of 100 patients were 52.4 ± 17.4 years, 63.4 ± 33.4 mins, 154.11 ± 67.6 mins, and 10.3 ± 5.7 days. The age difference of patients in all four groups (divided on the basis of type of liver parenchyma) was not statistically significant (Table 3). Patients of group A (normal liver parenchyma) had significantly less transection time in comparison to group B (cirrhotic livers) and group D (cholestatic livers), while it did not reach statistical difference when compared with group C (postchemotherapy livers) patients, though there was a trend towards lesser transection time in group A. This may be due to less number of patients in group C. The total operative time was significantly less in group A patients in comparison to group B, C, and D patients. Also group A patients had significantly less postoperative hospital stay in comparison with group B, C, and D patients. These results show that type of liver parenchyma affects the transection time, total operative time, and postoperative recovery as reflected by the postoperative hospital stay (Table 4).

Also it was found that the transection time and total operative time in Group 1 (first 50 patients) were significantly more than in Group 2 (second 50 patients), signifying the effect of surgeon's experience on it. Though, the postoperative hospital stay was similar in both these groups (Table 5).

Total 11/100 (11%) patients needed perioperative blood transfusions, with the range of 2–4 units per case. These included 1 patient of hydatid disease, 2 of secondaries liver, 1 of hilar cholangiocarcinoma, 3 of gallbladder cancer, 3 of HCC, and 1 patient of hepatoblastoma. Total 14/100 (14%) patients developed 22 postoperative complications (Table 6). Four patients had bile leak, ten had ascites, and five had wound infections.

Bile leak occurred in each patient after left hemihepatectomy for hydatid, right trisectionectomy for gallbladder cancer, right hemihepatectomy with segment I resection for hilar cholangiocarcinoma, and cystopericystectomy for hydatid cyst, with daily amount of 50 mL, 100 mL, 90 mL, and 20 mL, respectively. All but cystopericystectomy patient had preoperative biliary stent placement for obstructive jaundice. In all these patients it stopped conservatively in 9, 5, 6, and 2 days, respectively.

Ascites was seen in 5 HCC patients, 4 gallbladder cancer patients (with jaundice), and 1 cholangiocarcinoma (with jaundice) patient, with the hospital stay ranging from 15 to 26 days. It was managed successfully by fluid restriction, diuretics, bed rest, and low-salt diet.

All patients with wound infections had preoperative biliary stent placement. All of these had Gram-negative organisms and were successfully managed conservatively with dressings and antibiotics based on cultures of bile taken during surgery.

There were three mortalities due to hepatic encephalopathy, liver failure, and disseminated intravascular coagulation (DIC) in patients with HCC, gallbladder cancer, and hepatoblastoma, respectively.

### 3.1. Qualitative Ergonomic Evaluation

Debakey forceps has some differences over Kelly clamp on the basis of its design. Kelly clamp has a hinge in the middle with two finger loops which are grasped by the thump and ring finger, while the index finger helps guide the instrument. On the other hand, Debakey forceps are held between thumb and the index finger with top end resting on the first dorsal interosseous muscle at the base of the thumb and index finger. Spring tension at one end holds the grasping ends apart until pressure is applied. This allows one to quickly and easily grasp small tissue and to grasp and hold tissue easily with variable pressure [17]. It is less traumatic due to its fine tip and gentle enough to fracture only the liver parenchyma without injuring the ducts or vessels. Long and angled Debakey forceps with fine tip facilitates crushing in the deeper planes of liver. There is a definite sensation of tissue being crushed while using Debakey forceps, which thus helps in releasing the pressure timely thus preventing injury to vessels.

In present study, Debakey thumb forceps is found to have similar ergonomic advantages over Kelly clamp during crushing of liver parenchyma, as was reported in one study [15] when they were compared for their usage for diathermy. These advantages are that (1) a ringed handled instrument is much more difficult to pick up from a flat surface than thumb forceps like Debakey forceps as like many surgeons we like to pick them ourselves due to the involved repetitive movements of this kind, (2) the grip between the thumb and the side of the index finger for picking up thumb forceps required less accurate placing of the hand than putting the two digits through the finger loops of Kelly clamps which can be done without having to take focus away from the area of dissection, and (3) thumb forceps are held in the classical precision grip [18] in which the ulnar digits help in supporting the instrument between thumb and the index finger in addition to the apex of the thumb thus increasing the accuracy of handling, whereas the hand is unsupported while using the Kelly clamp.

**Figure 1:**
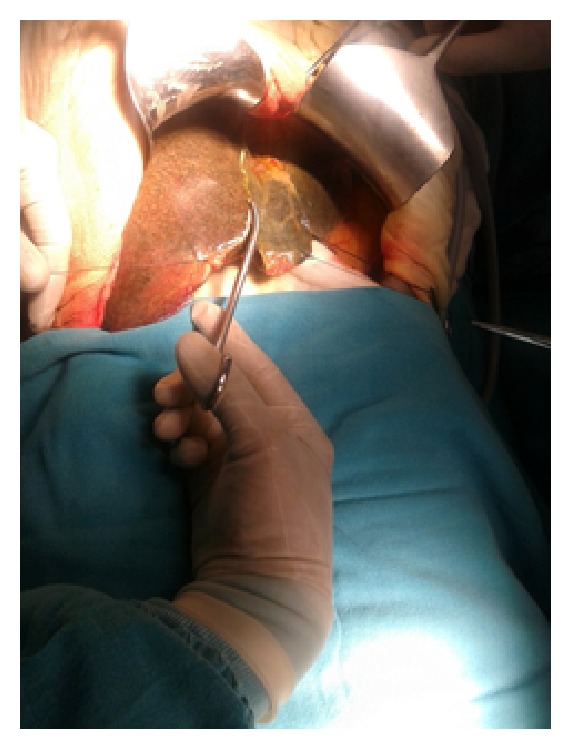
Abnormal posture of wrist while using Kelly clamp.

**Figure 2:**
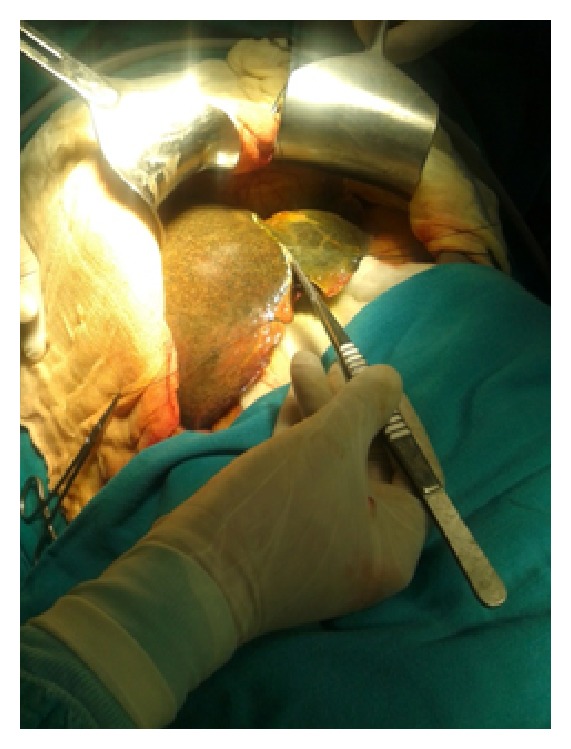
Neutral posture of wrist while using Debakey forceps.

**Figure 3:**
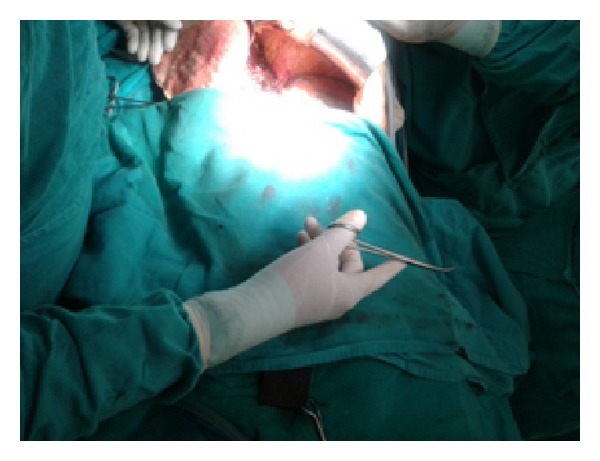
Position of Kelly clamp with neutral posture of wrist.

**Figure 4:**
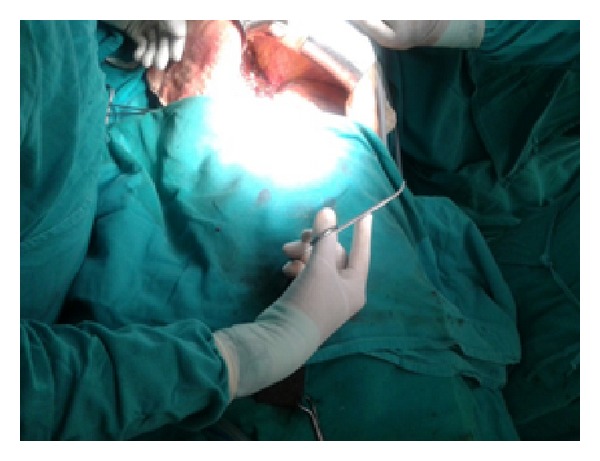
Position of Kelly clamp with slight flexion of wrist.

**Figure 5:**
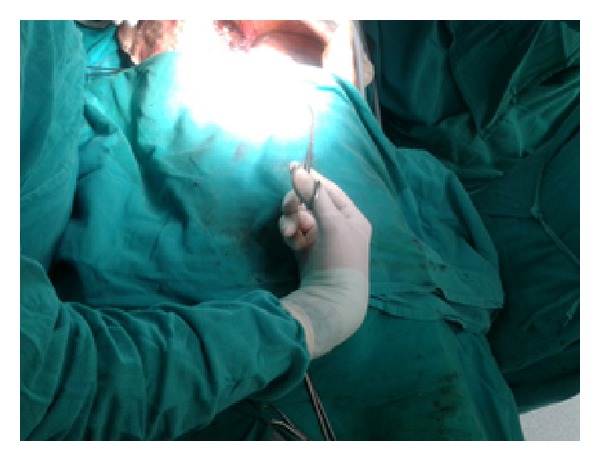
Functional position of Kelly clamp with awkward posture of wrist.

**Figure 6:**
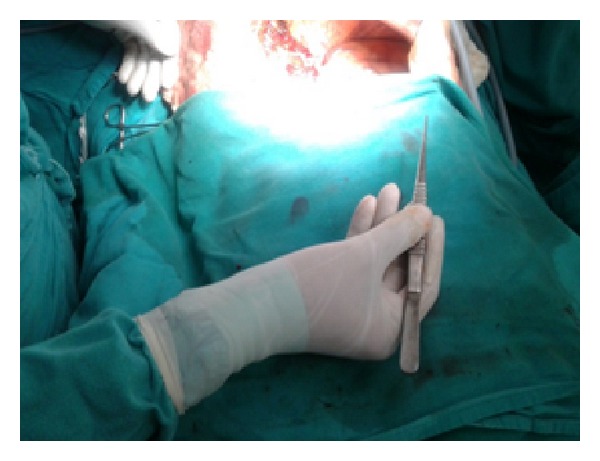
Position of Debakey forceps with neutral posture of wrist.

**Figure 7:**
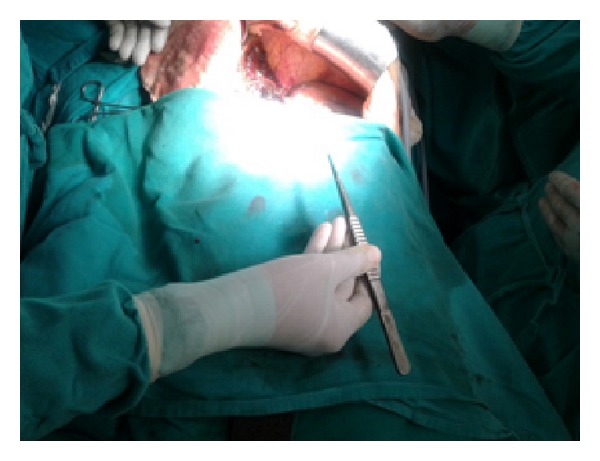
Inward position of Debakey forceps with neutral posture of wrist.

**Figure 8:**
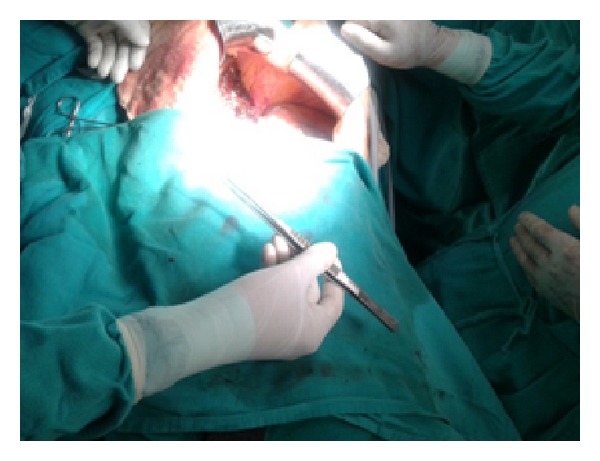
More inward position of Debakey forceps with neutral posture of wrist.

Figures 1–8 (photographs) depict wrist joint postures during liver parenchyma transection while using Kelly clamp and Debakey forceps. It is clear in Figures 2, 6, 7, and 8 that the wrist joint always remains in neutral posture during liver parenchyma transection with Debakey forceps at various depths and angles. On the contrary Figures 1, 3, 4, and 5 shows that wrist joint is always in an awkward and strainful posture while using Kelly clamp for liver parenchyma transection at all the depths and angles.

## 4. Discussion

This study shows that use of Debakey forceps crushing technique is safe and effective for liver parenchymal transection; transection time and total operative time improve with surgeon experience and it has ergonomic advantages over Kelly clamp technique.

The better understanding of liver anatomy and technical developments has helped in reducing the morbidity and mortality after liver resections [19–21]. Bleeding is the most important determinant of operative outcome after liver resection. Intraoperative blood loss with subsequent need for blood transfusion is significant risk factor for increased complication rates, poor postoperative outcomes, and shorter disease-free survival [22, 23]. Thus it is paramount to decrease the intraoperative blood loss and subsequent blood transfusions during liver resections. As most of the bleeding occurs during parenchymal transection of liver there are many methods devised from time to time to facilitate liver transection with minimal blood loss [9, 10].

Meta-analysis of 7 RCT with total 554 patients [24] has shown that there were no clinically important benefits of an alternative transection method in terms of blood loss, parenchymal injury, transection time, and hospital stay over clamp crushing method. So clamp crushing method remains the reference technique for transection of the parenchyma in elective hepatic resections. Also the 2009 Cochrane review [25] of randomized data failed to show any significant difference with regard to mortality, morbidity, and hospital stay while comparing clamp crushing technique to alternative methods. The clamp crushing avoids special equipment with similar or faster transection speed thus making it the most cost-effective technique which is 2 to 6 times cheaper than other methods depending on the number of surgeries performed each year [9, 24–28].

Our study shows that Debakey clamp is an equally effective instrument for parenchymal transection in all kinds of livers in terms of transection time and safety as is shown in previous reports using Kelly clamp technique [14]. The mean transection time in our study in normal liver was 48.1 ± 20.1 mins and it was significantly shorter than groups with patients with cirrhotic and cholestatic livers. The mean total operative time in our patients with normal liver was 110.4 ± 35.3 mins which was significantly shorter than groups with cirrhotic, postchemotherapy, and cholestatic livers.

The transfusion requirement in present study was 11% which is due to the inclusion of patients with all kinds of liver parenchyma. None of the patients with normal livers (group A) had blood transfusion which is similar to previous reports [14].

The mean postoperative length of hospital stay in subjects with normal livers was 10.3 ± 5.7 days which is similar to previous reports [9, 14, 28]. Subjects with diseased livers (groups B, C, and D) had greater hospital stay signifying the role of type of liver parenchyma on overall outcomes.

The morbidity rate in the present study is 14% (22 complications in 14 patients). Out of these 22 complications 21 have occurred in patients having cirrhotic, cholestatic, and postchemotherapy livers, while only one occurred in a patient of hydatid cyst with normal liver. The mortality in the present study is 3% with one patient each in cirrhotic, cholestatic and postchemotherapy liver groups.

All these results signify the importance of type of liver parenchyma on the transection time, total operative time, blood transfusion rates, morbidity, mortality, and postoperative hospital stay while using Debakey crush technique for liver resections.

The mean transection time and total operative time were found to be significantly more in the initial 50 cases of the total 100 cases suggesting the effect of surgeon's experience, though it did not affect the postoperative length of hospital stay (Table 5).

Technically, Debakey forceps has many advantages of Kelly clamp [14] including its efficacy and safety in all kinds of livers. It is also a cost-effective technique [25]. Ergonomically, there are two aspects which make Debakey forceps a preferred instrument compared to Kelly clamp for liver parenchymal crushing. One is the design of the instrument and the other is the posture of the wrist joint of the surgeon while operating with these instruments as described and shown above (Figures 1–8). These make Debakey forceps more useful to the operating surgeon in terms of easy handling, precise grip, ease of usage in every depth of liver resection, being less traumatic for the tissues, and giving least strain to the wrist joint by keeping it in the neutral position. This is because in neutral posture muscles are near their resting length thus making joints comfortable. For wrist joint it is neutral when forearm, wrist, and hands are all straight and in one line [29]. Awkward postures occur when wrist is in flexion or extension [30, 31]. In awkward posture muscles and ligaments of joint are either stretched or compressed. Thereby fatigue will occur more quickly, increasing the risk for injury [31].

Limitations of the present study include a nonrandomised trial design. However, this study is a single surgeon experience in consecutive cases and a large sample size with careful collection of data and is therefore important. We also performed a qualitative study of comparison of Debakey with Kelly technique and the findings are important.

In conclusion, this is the first such study showing that Debakey forceps crushing technique is as safe and effective method for liver parenchymal transection in all kinds of liver parenchyma with comparable results to Kelly clamp crushing method. It also shows that type of liver parenchyma has a significant effect on overall outcome while using Debakey crushing technique. Surgeon's experience is important. The technical and ergonomic differences between Debakey forceps and Kelly clamp, in terms of design and wrist joint posture, make Debakey forceps the preferred crushing technique for liver transection although large randomised trials are needed to confirm our findings. We therefore recommend Debakey forceps technique as the crushing method of choice for liver transection in elective liver resection operations in nontransplant setting. The ergonomic virtues of Debakey forceps should be considered while designing newer techniques and instruments for liver transection, especially in open liver resections.

## Figures and Tables

**Table 1:** Indications of liver resection in the study.

Indications	Numbers
Gallbladder cancer	32
Secondaries liver	15 (colorectal-11, GIST-04)
Hydatid disease	11
Hilar cholangiocarcinoma	09
Hemangioma	09
Hepatocellular carcinoma	08 (Child's A status)
Hepatoblastoma	04
Neuroendocrine tumor	02
Intrahepatic cholangiocarcinoma	02
FNH	02
Hamartoma	02
Recurrent pyogenic cholangitis	01
Hemangioendothelioma	01
Cancer hepatic flexure with local invasion of liver	01
Strictured hepaticojejunostomy with atrophy of right lobe liver with recurrent cholangitis	01
Total	**100**

**Table 2:** Types of liver resections.

Type of resection	Numbers
Right hemihepatectomy	19
Right hemihepatectomy with segment I resection	08
Left hemihepatectomy	09
Left hemihepatectomy with segment I resection	01
Right trisectionectomy	11
Left lateral sectionectomy	09
Segment IVb V resection	08
Segments IV, V, and VI resection	01
Segments V, VI, and VII resection	02
Segments V and VI resection	02
Segment III resection	01
Segment V resection	01
Segment VI resection	01
Wedge resection for gallbladder cancer	13
Right posterior sectionectomy	02
Cystopericystectomy	10
Nonanatomical resection	02
Total	**100**

**Table 3:** Baseline characteristics in different groups.

Characteristics	Group A (control) *N* = 53	Group B (cirrhosis) *N* = 8	Group C (CT) *N* = 14	Group D (cholestatic) *N* = 25
Sex				
Male (%)	24 (45.28)	8 (100)	9 (64.29)	20 (80)
Female (%)	29 (54.72)	0 (0.00)	5 (35.71)	5 (20)

Underlying diseases (%)				
Hypertension	7 (13.21)	3 (37.50)	1 (7.14)	0
DM	4 (7.55)	2 (25.00)	0	2 (8.00)
COPD	1 (1.89)	3 (37.50)	0	1 (4.00)
CAD	0	0	0	0

Control: normal liver parenchyma.

CT: postchemotherapy.

Cholestatic: obstructive jaundice.

DM: diabetes mellitus.

COPD: chronic obstructive pulmonary disease.

CAD: coronary artery disease.

**Table 4:** Comparison of age, transection time, total operative time, and postoperative hospital stay between four groups.

Characteristics	Group A (control) *N* = 53	Group B (cirrhosis) *N* = 8	Group C (CT) *N* = 14	Group D (cholestatic) *N* = 25	A versus B *P* value	A versus C *P* value	A versus D *P* value
Age (years)							
(Mean ± SD)	48.94 ± 16.27	57.25 ± 11.38	48.46 ± 22.93	47.32 ± 13.69	0.91	0.59	0.44
(Range)	23–85	40–70	4–71	25–77			
Transection time (minutes)							
(Mean ± SD)	48.09 ± 20.07	130.38 ± 44.38	60.64 ± 32.81	75.8 ± 19.72	0.00	0.07	0.00
(Median, IQR)	48, 21	145, 69.5	52.5, 64	69, 18			
(Range)	22–117	62–180	25–115	35–120			
Total operative time (minutes)							
(Mean ± SD)	110.41 ± 35.30	226.6 ± 57.8	145.21 ± 52.91	228.52 ± 43.20	0.00	0.004	0.00
(Median, IQR)	100, 32	218, 98.5	142.5, 94	209, 62			
(Range)	68–252	150–310	75–230	152–308			
Hospital stay (days)							
(Mean ± SD)	6.72 ± 1.85	14.62 ± 5.50	10.07 ± 5.50	16.52 ± 5.11	0.00	0.0004	0.00
(Median, IQR)	7, 3	14.5, 5	9, 414	16, 6			
(Range)	4–11	8–26	4–27	9–29			

Control: normal liver parenchyma.

CT: postchemotherapy.

Cholestatic: obstructive jaundice.

**Table 5:** Comparison of age, transection time, total operative time, and postoperative hospital stay in the first 50 and second 50 patients.

Characteristics	Group 1	Group 2	*P* value
	First 50 patients	Second 50 patients	
Age (years)			
(Mean ± SD)	48.08 ± 15.45	52.38 ± 17.44	0.52
(Range)	4–73	4.5–85	
Transection time (minutes)			
(Mean ± SD)	70.2 ± 31.02	56.52 ± 34.54	0.039
(Median, IQR)	65, 33	50.5, 34	
(Range)	25–160	22–180	
Total operative time (minutes)			
(Mean ± SD)	168.04 ± 66.50	140.20 ± 66.40	0.038
(Median, IQR)	155, 117	105, 100	
(Range)	68–305	74–310	
Hospital stay (days)			
(Mean ± SD)	9.66 ± 5.40	10.88 ± 5.95	0.28
(Median, IQR)	8.5, 7	8, 7	
(Range)	4–29	5–27	

**Table 6:** Postoperative complications.

Complication	Type of resection	Numbers	Disease
Bile leak	Left hemihepatectomy	01	Hydatid disease
	Cystopericystectomy	01	Hydatid disease
	Right trisectionectomy	01	Gallbladder cancer
	Right hemihepatectomy plus segment I resection	01	Hilar cholangiocarcinoma

Ascites	Right trisectionectomy	04	Gallbladder cancer
	Left hemihepatectomy plus segment I	01	Hilar cholangiocarcinoma
	Right hemihepatectomy	05	HCC

Wound infection	Left hemihepatectomy	01	Hydatid disease
	Right trisectionectomy	02	Gallbladder cancer
	Right hemihepatectomy plus segment I	01	Hilar cholangiocarcinoma
	Right hemihepatectomy	01	HCC

Hepatic encephalopathy*	Right hemihepatectomy	01	HCC

Postoperative liver failure*	Right trisectionectomy	01	Gallbladder cancer

DIC*	Right hemihepatectomy	01	Hepatoblastoma

*Signifies mortality.

## References

[1] Gozzetti G, Mazziotti A, Grazi GL (1995). Liver resection without blood transfusion. *British Journal of Surgery*.

[2] Cunningham JD, Fong Y, Shriver C, Melendez J, Marx WL, Blumgart LH (1994). One hundred consecutive hepatic resections: blood loss, transfusion, and operative technique. *Archives of Surgery*.

[3] Jarnagin WR, Gonen M, Fong Y (2002). Improvement in perioperative outcome after hepatic resection: analysis of 1,803 consecutive cases over the past decade. *Annals of Surgery*.

[4] Lin TY, Tsu K, Mien C, Chen C (1958). Study on lobectomy of the liver. *Journal of the Formosan Medical Association*.

[5] Bismuth H (1982). Surgical anatomy and anatomical surgery of the liver. *World Journal of Surgery*.

[6] Lin TY (1974). A simplified technique for hepatic resection: the crush method. *Annals of Surgery*.

[7] Lin TY (1973). Results in 107 hepatic lobectomies with a preliminary report on the use of a clamp to reduce blood loss. *Annals of Surgery*.

[8] Launois B, Jamieson GG, Starzl TE (1993). *Modern Operative Techniques in Liver Surgery*.

[9] Lesurtel M, Selzner M, Petrowsky H, McCormack L, Clavien P-A (2005). How should transection of the liver be performed? A prospective randomized study in 100 consecutive patients: comparing four different transection strategies. *Annals of Surgery*.

[10] Aragon RJ, Solomon NL (2012). Techniques of hepatic resection. *Journal of Gastrointestinal Oncology*.

[11] Schemmer P, Friess H, Hinz U (2006). Stapler hepatectomy is a safe dissection technique: analysis of 300 patients. *World Journal of Surgery*.

[12] Imamura H, Seyama Y, Kokudo N (2003). One thousand fifty-six hepatectomies without mortality in 8 years. *Archives of Surgery*.

[13] Sun HC, Qin LX, Lu L (2006). Randomized clinical trial of the effects of abdominal drainage after elective hepatectomy using the crushing clamp method. *British Journal of Surgery*.

[14] Kim KH, Lee SG (2008). Usefulness of Kelly clamp crushing technique during hepatic resection. *HPB*.

[15] Patkin M (1971). Ergonomics of diathermy forceps design. *Medical Journal of Australia*.

[16] Strasberg SM (2005). Nomenclature of hepatic anatomy and resections: a review of the Brisbane 2000 system. *Journal of Hepato-Biliary-Pancreatic Surgery*.

[17] Carlisle RP (2004). *Scientific American Inventions and Discoveries: All the MilesTones in Ingenuity—From the Discovery of Fire to the Invention of the Microwave Oven*.

[18] Patkin M (1967). Ergonomic aspects of surgical dexterity. *Medical Journal of Australia*.

[19] Rees M, Plant G, Wells J, Bygrave S (1996). One hundred and fifty hepatic resections: evolution of technique towards bloodless surgery. *British Journal of Surgery*.

[20] Doci R, Gennari L, Bignami P (1995). Morbidity and mortality after hepatic resection of metastases from colorectal cancer. *British Journal of Surgery*.

[21] Belghiti J, Hiramatsu K, Benoist S, Massault PP, Sauvanet A, Farges O (2000). Seven hundred forty-seven hepatectomies in the 1990s: an update to evaluate the actual risk of liver resection. *Journal of the American College of Surgeons*.

[22] Fong Y, Fortner J, Sun RL, Brennan MF, Blumgart LH (1999). Clinical score for predicting recurrence after hepatic resection for metastatic colorectal cancer: analysis of 1001 consecutive cases. *Annals of Surgery*.

[23] Rosen CB, Nagorney DM, Taswell HF (1992). Perioperative blood transfusion and determinants of survival after liver resection for metastatic colorectal carcinoma. *Annals of Surgery*.

[24] Rahbari NN, Koch M, Schmidt T (2009). Meta-analysis of the clamp-crushing technique for transection of the parenchyma in elective hepatic resection: back to where we started?. *Annals of Surgical Oncology*.

[25] Gurusamy KS, Pamecha V, Sharma D, Davidson BR (2009). Techniques for liver parenchymal transection in liver resection. *Cochrane Database of Systematic Reviews*.

[26] Rau HG, Wichmann MW, Schinkel S (2001). Surgical techniques in hepatic resections: ultrasonic aspirator versus jet-cutter. A prospective randomized clinical trial. *Zentralblatt fur Chirurgie*.

[27] Sakamoto Y, Yamamoto J, Kokudo N (2004). Bloodless liver resection using the monopolar floating ball plus ligature diathermy: preliminary results of 16 liver resections. *World Journal of Surgery*.

[28] Takayama T, Makuuchi M, Kubota K (2001). Randomized comparison of ultrasonic versus clamp transection of the liver. *Archives of Surgery*.

[29] Warren N, Morse TF Neutral posture. http://www.oehc.uchc.edu/ergo_neutralposture.asp.

[30] Keir PJ, Bach JM, Hudes M, Rempel DM (2007). Guidelines for wrist posture based on carpal tunnel pressure thresholds. *Human Factors*.

[31] Chaffin D, Andersson GBJ, Martin BJ (2006). *Occupational Biomechanics*.

